# Effect of follicle-stimulating hormone and luteinizing hormone levels on egg-laying frequency in hens

**DOI:** 10.14202/vetworld.2022.2890-2895

**Published:** 2022-12-20

**Authors:** Ragil Angga Prastiya, Sri Pantja Madyawati, Sera Yunita Sari, Aras Prasetiyo Nugroho

**Affiliations:** 1 Department of Veterinary Sciences Division of Veterinary Reproduction, Faculty of Veterinary Medicine, Universitas Airlangga, Surabaya, Indonesia; 2 Department of Reproduction, School of Health and Life Sciences (SIKIA), Universitas Airlangga, Banyuwangi, Indonesia; 3 Aves Vet. Professional Interest, Kediri, Indonesia; 4 Department of Animal Science, Jenderal Soedirman University, Purwokerto, Indonesia

**Keywords:** egg-laying frequency, enzyme-linked immunosorbent assay, gonadotropin hormone, laying hens, reproductive health

## Abstract

**Background and Aim:**

Gonadotropins, for example, follicle-stimulating hormone (FSH) and luteinizing hormone (LH), are hormones that affect the reproductive process. In hens, optimal levels of FSH and LH can stimulate follicle growth fairly rapidly and thereby increase egg production through follicle development and increased ovulation. Follicle-stimulating hormone acts in the early stages of follicular growth, whereas LH acts on pre-ovulatory follicles. Normal follicular growth is the result of the complementary action of FSH and LH. Low FSH and LH levels result in the formation of follicles but a lack of egg production in chickens. This study aimed to investigate FSH and LH hormone levels from layer chickens with different egg-laying frequencies.

**Materials and Methods:**

Fifty blood serum samples were collected from 54-week-old ISA brown strain hens that were divided into five groups (with 10 hens per group) as follows: Hens that lay eggs (i) every day, (ii) once every 2 days, (iii) twice every 3 days, (iv) 3 times every 4 days, and (v) hens that do not lay eggs. Follicle-stimulating hormone and LH levels were measured in samples using an enzyme-linked immunosorbent assay, and the data were analyzed using multivariate analysis of variance.

**Results:**

Follicle-stimulating hormone levels were significantly associated with the frequency of egg laying in ISA brown strain hens (p < 0.05); the highest FSH level (869.005 ± 149.194 pg/mL) was found in hens that lay eggs every day. In contrast, the highest LH level (51.386 ± 2.410 mIU/mL) was found in hens that lay eggs every 2 days.

**Conclusion:**

High level of FSH (869.005 ± 149.194 pg/mL) was associated with a high frequency of egg laying (every day) in ISA brown strain hens, and LH level of around 30.406 pg/mL was associated with daily egg laying in these hens.

## Introduction

Hens can produce eggs only once a day and some hens do not lay any eggs over several days. Furthermore, not all hens in a single cage will lay eggs at the same rate; that is, some hens never lay eggs, whereas some hens produce eggs earlier than others [1]. In addition, laying hens may stop laying eggs and some scenarios will cause a decrease in or end to egg laying [2]. Egg production is influenced by age as well as genetic and environmental factors, among other factors [3]. Environmental factors that affect egg production include lighting and temperature [4]. The age at which pullets mature sexually directly affects their egg-laying performance and genetic stocks have an optimal age at which they reach sexual maturity to ensure that they produce the maximum possible egg mass [5]. Among the factors affecting the time of sexual maturation, the lighting program (day length and light intensity) to which a flock of laying hens is subjected during the growth and production phases plays an important role [6]. Indeed, the lighting program affects the achievement of peak production because it is related to the maturation of reproductive organs [7]. Artificial light provided at night, especially in poultry, can stimulate the development of the reproductive system and accelerate genital maturation [8]. Poultry also experience early molting under night conditions without the addition of light [9].

Long-wave light more easily penetrates the skin tissue to stimulate the secretion of hormones that control reproduction from the pituitary gland [10]. These hormones include gonadotropins such as follicle-stimulating hormone (FSH), which stimulates the development and maturity of the ovum, and luteinizing hormone (LH), which plays a role in the ovulation of the ovum [11]. Optimal levels of FSH and LH can stimulate the growth of follicles fairly rapidly, resulting in increased egg production due to the increased number of follicles developed and ovulated [12]. The control of the flow of such hormones in poultry is unique; that is, the flow of gonadotropins is regulated not only in the largest follicles ready for ovulation but also to maintain the existence of the follicular hierarchy [13]. Normal follicular growth results from the complementary action between FSH and LH [14, 15].

This study aimed to investigate FSH and LH hormone levels from layer chickens with different egg-laying frequencies.

## Materials and Methods

### Ethical approval

All research procedures were performed following recommendations from the Local Experimental Animal Care Committee on Ethics, Airlangga University, Indonesia (number: 112/HRECC.FODM/III).

### Study period and location

The study was conducted during the hot-dry season from January to May 2022. The samples used in this study were obtained from a poultry farm (Saredo Jaya Abadi Farm) in Watugede Village, Puncu District, Kediri Regency, East Java, Indonesia (latitude: 7.816150; longitude: 112.174730; elevation: 195 m above sea level). Blood samples were tested for reproductive hormone levels at Gamma Scientific Biolab, Malang Regency, East Java, Indonesia.

### Meteorological data

A thermometer (Mason’s Hygrometer; GH Zeal Limited, London, UK) was placed in the poultry pen at the height of 1.5 m and close to the laying hens. The dry-bulb and wet-bulb temperatures were recorded every 4 h from 07:00 to 19:00 throughout the 8-week duration of the study.

### Animal selection

Laying ISA brown hens aged 54 weeks and with a mean live weight of 1.85 ± 0.3 kg were obtained from a breeding company (ISA North America, Ontario, Canada) and used in this study. The flock was given access to feed and water *ad libitum*, and their feed had the following nutritive values: 17.5% crude protein, 8.0% crude fiber, 5.0% crude fat, 35.0% ash, 4.0% Ca, 0.5% phosphor, and 2750 kCal/kg metabolizable energy. Standard routine prophylactic vaccinations and periodic medication were administered to the laying hens.

Observations were conducted for 14 days in advance of selecting hens for blood sampling. Each day, all hens were checked 3 times (morning, afternoon, and evening), and their cage was marked with a “1” if they had laid an egg or a “0” if they had not laid an egg. Hens were selected for blood sampling if they exhibited a minimum repetition of two egg-laying cycles.

**Table:** 

101010101	111011110	011101110	011011001
T2 sample	X	T4 sample	X

An example of hen selection for blood sampling is given above. The hen marked “T2 sample” exhibited a regular egg-laying frequency, that is, laying an egg every other day. In contrast, the hen marked X mark exhibited an irregular egg-laying frequency and was not chosen for sampling. Hens that were chosen for sampling were themselves marked to facilitate blood sampling.

### Experimental design and animal management

A completely randomized design was used in this study. Fifty hens were divided into five groups as follows:

- T1: Hens that lay eggs every day.
- T2: Hens that lay eggs once every 2 days.
- T3: Hens that lay eggs twice every 3 days.
- T4: Hens that lay eggs three times every 4 days.
- T5: Hens that do not lay eggs.

Layer hens were housed in a conventional cage in a closed house and fed the common standard diet during the research period. The temperature of the laying hen room was controlled at 26°C ± 3°C, and a 16: 8 h light: dark cycle was used. The egg-laying frequency of the hens was recorded for 14 days, and blood samples were taken after determining egg-laying frequency.

Blood samples (1 mL) were obtained from the brachial vein of each hen’s wing and stored in a vacutainer plain tube in a cool box (4°C) filled with ice gel and ice cubes. The standard for the delivery of blood samples was adjusted to the Decree of the Minister of Health of the Republic of Indonesia (number 1406/MENKES/SK/XI/2002) after the samples were collected and stored as described above.

### Follicle-stimulating hormone and LH levels

An enzyme-linked immunosorbent assay (ELISA) was used to determine the FSH and LH levels in hen blood samples; in the ELISA, the tested samples competed with antibodies contained in the microplate. Reagents or unbound materials were removed after leaching, and a substrate was added to the microplate, leading to color formation at the bond between the antibodies and conjugate enzymes. The optical density (OD) was then measured using an ELISA reader [16].

### Statistical analysis

Follicle-stimulating hormone and LH levels were analyzed using analysis of variance using SPSS 20 software (IBM Corp., NY, USA). If a significant difference was found at p < 0.05, Duncan’s test was used to determine the significant group.

## Results

### Follicle-stimulating hormone levels

**Figure-1:**
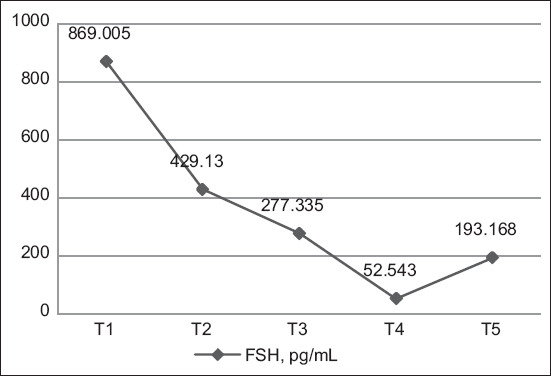
Activity of follicle-stimulating hormone levels.

**Figure-2:**
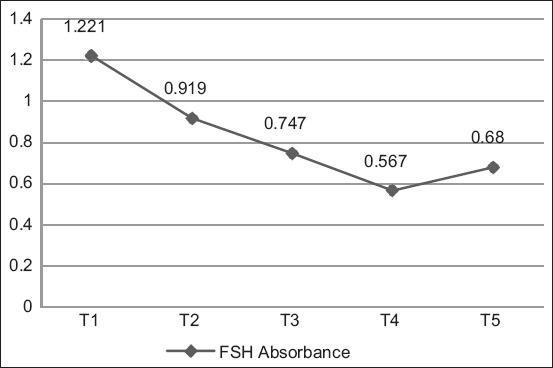
Average absorbance values of follicle-stimulating hormone level measurements.

The highest average FSH levels were found in the hens who laid an egg every day (T1: 869.005 pg/mL), whereas the lowest FSH levels were found in the hens who laid eggs for three times every 4 days (T4: 52.543 pg/mL) (Figure-1). The highest absorbance value was found in hens that laid an egg every day (T1: 1.221), whereas the lowest absorbance value was found in hens that laid eggs for three times every 4 days (T4: 0.567) (Figure-2). According to Duncan’s test results, the FSH levels and absorbance values of the T1 hen group were significantly higher than those of the other hen groups.

**Figure-3:**
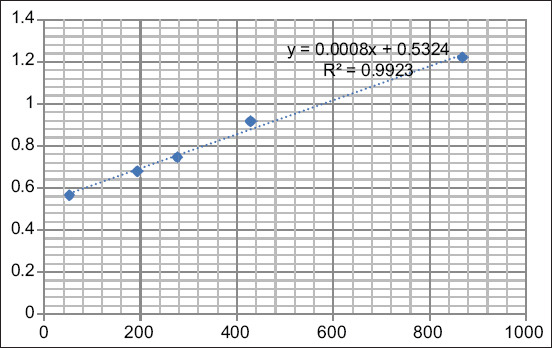
Calculation of the follicle-stimulating hormone standard curve.

The absorbance value is used to measure a sample’s reactivity. When the color produced is intense, the OD read will be high, indicating a high number of analytes. The FSH standard curve is presented in Figure-3. The R^2^ value was 0.9699, indicating that the curve had an accuracy of 96.99%.

### Luteinizing hormone levels

**Figure-4:**
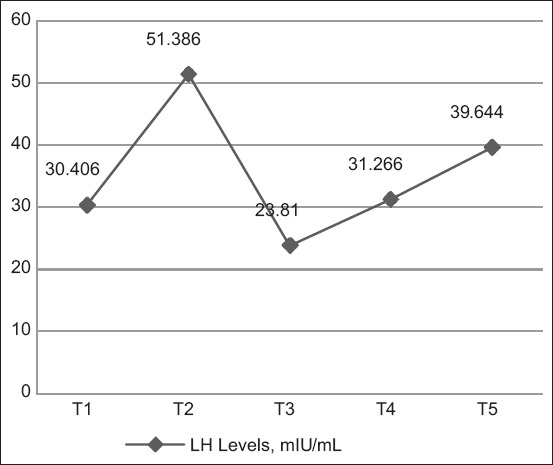
Activity of luteinizing hormone levels.

**Figure-5:**
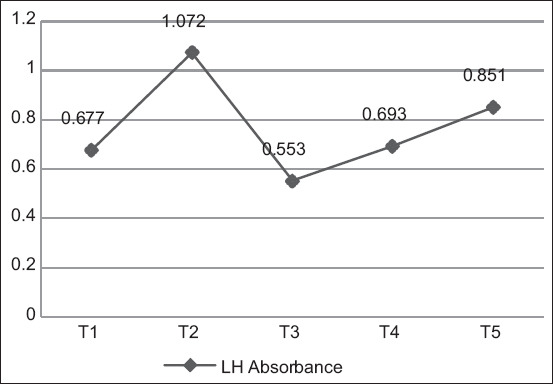
Average absorbance values of luteinizing hormone level measurements.

The highest average LH levels were found in hens who laid eggs once every 2 days (T2: 51.386 mIU/mL), whereas the lowest LH levels were found in hens who laid eggs twice every three days (T3: 23.810 mIU/mL) (Figure-4). The highest absorbance value was found in hens who laid eggs once every 2 days (T2: 1.072), whereas the lowest absorbance was found in hens who laid eggs twice every three days (T3: 0.553) (Figure-5). Duncan’s test results indicated that the hormone levels of T1 hens differed markedly from T2, T3, and T5 hens but not T4 hens. In addition, Duncan’s test revealed that LH levels were the highest in hens that laid eggs once every 2 days (T2).

**Figure-6:**
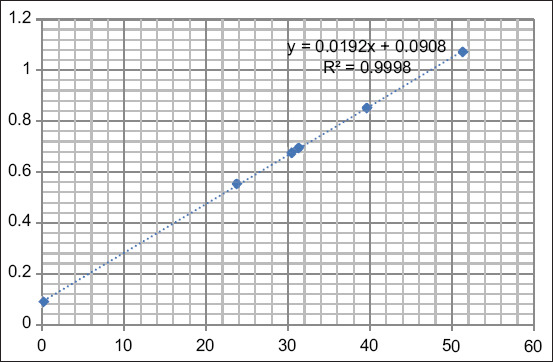
Calculation of the luteinizing hormone standard curve.

Absorbance was read using an ELISA reader tool with measurements at 450 nm. The antigen-antibody activity was expressed according to the OD measured after the substrate was added. The LH calibration curve is shown in Figure-6. The R^2^ value was 0.9752, indicating that the accuracy of the measured LH level was 97.52%.

## Discussion

Follicle-stimulating hormone plays an important role in the fertility rate of male and female animals as well as reproductive physiology [17]. It is produced in the anterior pituitary in response to gonadotropin-releasing hormone (GnRH) from the hypothalamus. In hens, FSH is responsible for the recruitment and development of granulosa cells from small follicles [18]. It acts mainly on the granulosa layer of small yellow follicles and acts on large and small follicles [19]. The reproductive hormonal status of a hen affects its egg production and quality [20]. Follicle-stimulating hormone improves follicular development by increasing angiogenesis in the theca externa follicles of laying hens with low egg production rates [21].

This results indicated that average FSH levels were the highest (869.005 pg/mL) in ISA brown hens that lay eggs every day (T1). Hens that laid eggs every 2 days (T2) exhibited lower average FSH levels (429.130 pg/mL), and FSH levels were lower still in hens that laid eggs for twice every three days (T3) (277.335 pg/mL). Hens that laid eggs for twice every three days (T4) exhibited significantly lower average FSH levels (52.543 pg/mL) than the other hen groups. Hens that did not lay eggs (T5) exhibited higher average FSH levels (193.168 pg/mL) than T4 hens but lower levels than T3 hens. The higher FSH levels in T5 hens relative to T4 hens were likely due to the T5 hens experiencing an egg-laying rest period. Old laying hens that do not lay eggs are known to have higher FSH levels than hens that lay eggs [22]. Follicle-stimulating hormone levels increase during a rest period after a stress period; in a previous study, FSH levels did not differ substantially from those measured at the peak of production [23].

Follicle-stimulating hormone is produced by the anterior pituitary gland, and the secretion of FSH is induced by GnRH secreted by the hypothalamus [24]. Follicle-stimulating hormone is an important hormone in the folliculogenesis of the ovaries and in steroidogenesis. Indeed, FSH plays a critical role in the maturation and differentiation of follicles. It also stimulates the production of progesterone hormones and steroid hormones, as well as the proliferation and differentiation of granulosa cells [25, 26]. Ovaries with a large number of follicles tend to have high FSH levels [27]. Follicle-stimulating hormone affects the steroidogenesis of immature yolk follicles and small yellow follicles but not large follicles in pre-ovulation (F1) [28]. Follicle-stimulating hormone not only improves the differentiation of granulosa cells in pre-hierarchical follicles but also facilitates the synthesis of steroid hormones in granular cells [15]. In hens with low egg production, a decrease in the number of pre-hierarchical follicles is partly due to an increase in the number of atretic follicles; atretic follicles exhibit fewer blood vessels and a decrease in the expression of FSH and FSH receptors [21].

A decrease in FSH levels can occur due to increased levels of prolactin at high concentrations exceeding those required in the egg-making process. The highest levels of prolactin affect the inhibition of GnRH secretion, leading to the inhibition of egg production [14]. Light is an external factor that influences the hypothalamus and triggers the stimulation of GnRH [7]. The lighting program affects the reproductive organs of laying hens, leading to the secretion of gonadotropin hormones such as FSH and LH [29]. Photoreceptors are responsible for the vision of laying hens, and the non-visual (extraretinal) photoreceptor is responsible for detecting the photoperiod, leading to the adjustment of the hen’s physiology in response to their environment [30]. The color of light is determined by its wavelength [31] and red light can stimulate an increase in the concentrations of FSH and LH [32].

Melatonin biosynthesis in both the pineal gland and retina is controlled by light. Melatonin is secreted at night and the presence of light at night can suppress melatonin synthesis [33]. An increased concentration of melatonin can increase the secretion of gonadotropin inhibitory hormone (GnIH), which indirectly reduces the secretion of GnRH, which, in turn, increases the concentration of GnIH in plasma and influences the inhibition of the reproductive axis [34], further reducing egg production [7].

Luteinizing hormone is secreted in the anterior pituitary and sent to the gonads, where it affects the release of testosterone from the testicles or estrogen from the ovaries. Luteinizing hormone plays an important role in inducing steroidogenesis and follicular development [35]. It also controls ovulation, with the highest secretion of LH occurring 6 h before ovulation [36]. Luteinizing hormone affects the production of follicular growth factors and hormones inside the ovaries [28]. As well as ovulation, the release of LH can stimulate the development of the ovaries, the secretion of progesterone and steroids and the rupture of the stigma of the ovum [37].

This results indicated that ISA brown hens that lay eggs every day (T1) had an average LH level of 30.406 mIU/mL, compared with the average LH level of T2 hens, that of hens that laid eggs every 2 days (T2) was significantly higher at 51.386 mIU/mL, which was the highest average LH level found in this study. The average LH level of hens that laid eggs for twice every 3 days (T3) was 23.810 mIU/mL, which was significantly lower than that of T2 hens and the lowest average LH level found in this study. Hens that laid eggs for 3 times every 4 days (T4) exhibited a higher LH level (31.266 mIU/mL) than T3 and T1 hens. The LH level of hens that did not lay eggs (T5) was 39.644 mIU/mL, which was higher than that of T1, T3, and T4 hens.

In this study, LH levels fluctuated among the groups of hens, likely because LH levels had not peaked at the time of blood sampling. Luteinizing hormone concentrations peak 4–6 h before ovulation, whereas the lowest LH concentrations are observed about 11 h before ovulation [22]. Blood was sampled from the hens in this study simultaneously, regardless of the timing of egg production; therefore, it is possible that T1 hens had a lower average LH level than T2, T4, and T5 hens, even though their egg production was lower than that of T1 hens.

Luteinizing hormone is secreted by the anterior pituitary due to an increase in the progesterone synthesized by the pre-ovulatory follicles. The increase in progesterone synthesis is caused by a decrease in estrogen hormone levels, which is due to an increase in the size of the follicles in the follicular hierarchy, especially the largest follicles (F1) [7]. Luteinizing hormone is secreted due to positive feedback from progesterone secreted by the pre-ovulation follicles [36]. The excessive injection of pituitary extract increases estrogen and progesterone levels, causing negative feedback on LH secretion that reduces LH levels and hampers the ovulation process, which, in turn, reduces egg production [8].

The final reproduction period increases the LH surge interval and its variability. Animals experience a decrease in the growth rate of ovarian follicles, and the time required for ovarian follicles to mature into F1 is increased [38]. Light is an environmental factor that stimulates the secretion of GnRH [7]. A photoperiod of 12 h light, 2 h dark, 4 h light, and 6 h dark is considered optimal for the egg-laying process, likely due to the optimal concentrations of prolactin and LH produced during this photoperiod [39]. Ambient temperature is an environmental factor that causes a decrease in egg production. High ambient temperature leads to heat stress in hens, which affects the appearance and health of the hen. Indeed, heat stress causes several physiological changes in laying hens, including an acid-base imbalance, the suppression of immunocompetence increasing risk of mortality, and decreased feed efficiency, body weight, and egg production [40].

The data in our study suggest that laying hens that experience the same photoperiod show differences in FSH and LH levels and egg-laying frequency. Thus, in relation to laying hens, improved monitoring of the equitable distribution of lighting, equitable distribution of nutrients, and pullet quality are required.

## Conclusion

Based on the results of the study, a higher FSH level is associated with a higher frequency of egg laying in ISA brown hens. In addition, an LH level of 30.406 mlU/mL is sufficient for ISA brown hens to lay eggs once per day.

## Authors’ Contributions

RAP and SPM: Conceived the study design. SYS and APN: Identified the reproductive hormonal status and collected blood samples. RAP: Drafted the manuscript. SPM: Revised the manuscript. All authors have read and approved the final manuscript.
